# Saliva samples are a viable alternative to blood samples as a source of DNA for high throughput genotyping

**DOI:** 10.1186/1755-8794-5-19

**Published:** 2012-05-30

**Authors:** Jean E Abraham, Mel J Maranian, Inmaculada Spiteri, Roslin Russell, Susan Ingle, Craig Luccarini, Helena M Earl, Paul PD Pharoah, Alison M Dunning, Carlos Caldas

**Affiliations:** 1 Department of Oncology and Strangeway’s Research Laboratory, University of Cambridge, Cambridge, UK; 2 Cambridge Breast Unit and NIHR Cambridge Biomedical Research Centre, University of Cambridge NHS Foundation Hospitals, Hills Road, Cambridge, UK; 3 Cancer Research UK Cambridge Research Institute, Li Ka Shing Centre, Robinson Way, Cambridge, UK; 4 Cambridge Experimental Cancer Medicine Centre, Cambridge, UK

## Abstract

**Background:**

The increasing trend for incorporation of biological sample collection within clinical trials requires sample collection procedures which are convenient and acceptable for both patients and clinicians. This study investigated the feasibility of using saliva-extracted DNA in comparison to blood-derived DNA, across two genotyping platforms: Applied Biosystems Taqman^TM^ and Illumina Beadchip^TM^ genome-wide arrays.

**Method:**

Patients were recruited from the Pharmacogenetics of Breast Cancer Chemotherapy (PGSNPS) study. Paired blood and saliva samples were collected from 79 study participants. The Oragene DNA Self-Collection kit (DNAgenotek®) was used to collect and extract DNA from saliva. DNA from EDTA blood samples (median volume 8 ml) was extracted by Gen-Probe, Livingstone, UK. DNA yields, standard measures of DNA quality, genotype call rates and genotype concordance between paired, duplicated samples were assessed.

**Results:**

Total DNA yields were lower from saliva (mean 24 μg, range 0.2–52 μg) than from blood (mean 210 μg, range 58–577 μg) and a 2-fold difference remained after adjusting for the volume of biological material collected. Protein contamination and DNA fragmentation measures were greater in saliva DNA. 78/79 saliva samples yielded sufficient DNA for use on Illumina Beadchip arrays and using Taqman assays. Four samples were randomly selected for genotyping in duplicate on the Illumina Beadchip arrays. All samples were genotyped using Taqman assays. DNA quality, as assessed by genotype call rates and genotype concordance between matched pairs of DNA was high (>97%) for each measure in both blood and saliva-derived DNA.

**Conclusion:**

We conclude that DNA from saliva and blood samples is comparable when genotyping using either Taqman assays or genome-wide chip arrays. Saliva sampling has the potential to increase participant recruitment within clinical trials, as well as reducing the resources and organisation required for multicentre sample collection.

## Background

Incorporation of biological sample collection within clinical trials and cohort studies will enhance our ability to identify biomarkers that improve individualisation of treatments. This requires sample collection procedures which are convenient and acceptable for both patients and clinicians. Whilst blood sampling and tumour biopsies are essential in some circumstances, often a less invasive procedure is sufficient and would improve trial recruitment. The potential advantages of saliva sample collection compared with blood sample collection include lower overall cost, lower infection risk, increased patient convenience, acceptability, compliance and uptake. However potential disadvantages include lower mean DNA yield and greater contamination with bacterial DNA. This study investigates the suitability of DNA extracted from saliva compared with DNA extracted from blood for high-throughput genotyping platforms.

Buccal sampling as an alternative to venous blood sampling has been investigated previously, but there is still reluctance to using DNA extracted from saliva samples or buccal cyto-brushes [1-7]. The main reasons for this are concerns over reduced yield and quality of DNA extracted from saliva, especially given the rigorous DNA requirements for high-throughput technologies. Studies investigating bacterial content in saliva collection samples have estimated that the median bacterial content from Oragene saliva collection kits is 11.8%, whereas bacterial content from mouthwash and buccal swabs was up to 60% and 90% respectively [8]. One study (n = 23), using the Illumina HumanHAP300 beadchip, concluded that under optimal conditions DNA from buccal cells provided comparable results to blood-derived DNA [9]. Another study successfully used saliva-extracted DNA in a Genome Wide Association Study (GWAS), but there was no clear comparison of the mean DNA yield or range of DNA yield found with each sample type. However the study did show comparable concordance and call rates [10]. One published study in dogs compared the performance of paired canine saliva and blood-derived DNA using the Illumina Infinium platform. This study demonstrated that canine saliva DNA was suitable for high-throughput genotyping studies [11].

This study compares DNA extracted from blood and saliva across two genotyping platforms. The Applied Biosystems Taqman^TM^ platform allowed comparison of paired saliva and blood DNA samples in 79 study participants, while the Illumina beadchips^TM^ compared genotyping quality across thousands of SNPs in four participants.

The study also compared: (i) DNA yields from normal extraction procedures on 9 ml EDTA blood tubes (monovette/vacutainer) and Oragene DNA Saliva Self-Collection kits (DNA Genotek®) (ii) Ratios of Absorbance at 260 and 280 nm (iii) DNA fragmentation (iv) Genotype Call Rates (i.e. the number of results obtained) for each type of DNA and (v) Genotype Concordances between matched pairs of samples – a measure of the accuracy of the genotypes obtained.

## Methods

### Patient samples

Paired blood and saliva samples (n = 79) were collected from a subset of patients participating in the Pharmacogenetics of Breast Cancer Chemotherapy (PGSNPS) study, which recruited patients from four UK breast cancer chemotherapy studies [12-14]. After obtaining written, informed consent, two EDTA blood samples (median volume 8 ml) were initially collected from all participants.

Subsequently participants were randomly selected for the saliva-blood feasibility study. These participants were contacted by mail and received an information leaflet explaining the aims and requirements of the study, a consent form which the patient signed, if they wished to participate, or alternatively a form to decline entry into the study and a stamped addressed envelope. If the patient consented, a trial research nurse or doctor would co-sign the consent form, retain a copy centrally and return a copy to the patient, enclosing the Oragene DNA Self-Collection kit (DNA Genotek®) together with the manufacturer’s instructions. Eighty-five percent of patients approached regarding the saliva-blood feasibility study gave consent and entered the study. The self-collection method did not require the participation of a regional hospital to recruit the patient. Patients recruited to this feasibility study returned the samples by post or in person, over a period of 2 months from April 2008 to May 2008. Samples were received by a nominated research nurse who stored the samples at room temperature until the samples were brought to the laboratory for extraction. As samples were received at different time points, different samples will have been stored for variable lengths of time.

The saliva-blood feasibility study received ethical approval in April 2008 from the Cambridgeshire 1 (formerly Huntingdon) Research Ethics Committee, UK.

### Saliva DNA extraction

The Oragene saliva collection device was selected for use because at the time the study was initiated this was the most well-established and commonly used saliva collection system. DNA was extracted from a 500 μl aliquot from the Oragene DNA/saliva Self-Collection kits in accordance with the manufacturer’s instructions [15]. Each sample was bar-coded, and stored until required for DNA extraction. Extracted samples were stored at −20°C prior to genotyping. DNA was quantified using Picogreen (Invitrogen, Ltd), according to manufacturer’s instructions, which specifically measures double stranded DNA, thus ensuring more accurate measurement of the human DNA component of total saliva DNA. All samples were normalised to 2 ng/μl, based on the Picogreen quantification, prior to genotyping.

### Blood DNA extraction

Lymphocyte DNA has been extracted and normalised to 40 ng/μl for storage by Gen-Probe, Livingstone, UK, using a single-tube chloroform extraction procedure. Subsequently this DNA was normalised to 2 ng/μl for genotyping

### Taqman genotyping

Genotyping was performed using Taqman® according to manufacturer’s instructions. Primers and probes (FAM and VIC labelled) were supplied directly by Applied Biosystems as Assays-by-Design^TM^ (see Additional file 1: Figure S1). Saliva DNA samples were plated out in duplicate on a 384 well plate, alongside additional duplicate DNAs taken from the paired blood samples (all normalised to 2 ng/μl). Plates were read on the ABI Prism 7900 using the Sequence Detection Software (Applied Biosystems). Four assays, which had been previously used in other research projects (data not shown) and therefore known to work well, were chosen for this experiment.

### Genome wide genotyping of paired blood and saliva samples using HumanHap duo BeadChip arrays

DNA from four saliva samples was genotyped in duplicate using HumanHap300 Duo Illumina BeadChip arrays. DNA from matching blood samples was genotyped in duplicate using HumanHap550 Duo BeadChips. The change in array was due to the manufacturers stopping availability of HumanHap300 Duo Illumina BeadChip arrays, during the period the study was conducted. The assay was performed following manufacturer’s instructions [16] using 750 ng of each DNA sample, normalized to 50 ng/μl.

### Spectrophotometry to assess DNA purity

Ten matched saliva (of varying yields) and blood samples were assessed for A260/A280 ratio (Additional file 1: Table S1). A 1/100 dilution was prepared (DNA/deionised H_2_0) and analysed, using a CECIL CE2041 (2000 SERIES) spectrophotometer, according to the manufacturers instructions.

### Microchip electrophoresis to assess fragmentation of DNA

Three matched saliva and blood DNA samples were randomly selected and assessed (see Additional file 1: Figure S2) using the Shimadzu Microchip Electrophoresis System and SYBR®Gold nucleic acid gel stain, (2 μl DNA in 4 μl DNA-500 Marker) according to manufacturer’s instructions.

## Results

The yields of DNA extracted from blood and saliva, as measured by Picogreen (Invitrogen Ltd) are shown in Table 1. We aimed to collect 2 ml of saliva and 9 ml blood from each participant but not all provided ideal volumes. The actual volumes of saliva obtained were not recorded but approximate volumes of blood obtained from each participant were recorded (median 8 ml, range 4–10 ml). Total DNA yields from the saliva samples (mean 24 μg, range 0.2–52 μg) were lower than from the blood samples (mean 210 μg, range 58–577 μg) and an approximate 2-fold difference in yield remained after adjusting for the biological sample volume collected. However the yield from all but one of the saliva samples was sufficient for chip genotyping (chip genotyping protocols vary, but typically require 500 ng-1 μg DNA at concentrations of 50–100 ng/μl). The chips used in this study each required 750 ng DNA.

**Table 1:** DNA yields from blood and saliva

**Sample type**	**Sample volume collected (range)/ml**	**Mean DNA yield (range)/μg**	**Mean yield per ml sample collected (range)/μg**
**Blood**	**8 (4–10)**	**210 (58–577)**	**26 (6–73)**
**Saliva**	2*	**24 (0.2 - 52)**	**12 (0.1 - 26)**

*in 2 ml Oragene solution.

The quality of the DNA obtained was assessed by several criteria. Ratios of absorbance at 260 nm and 280 nm are presented in Additional file 1: Table S1. In this study DNA from blood has a mean (A260/A280 ratio) value (1.71), very close to the ideal value of 1.8. The mean (A260/A280 ratio) values from saliva is a little lower (1.56), indicating more protein contamination in the saliva extracted DNA. DNA fragmentation was compared on a bio-analyser and the results are shown in Additional file 1: Figure S2. Both DNA types have a clear high molecular weight band, but a more intense band of fragmented DNA is visible in DNA extracted from saliva.

**Figure 1:**
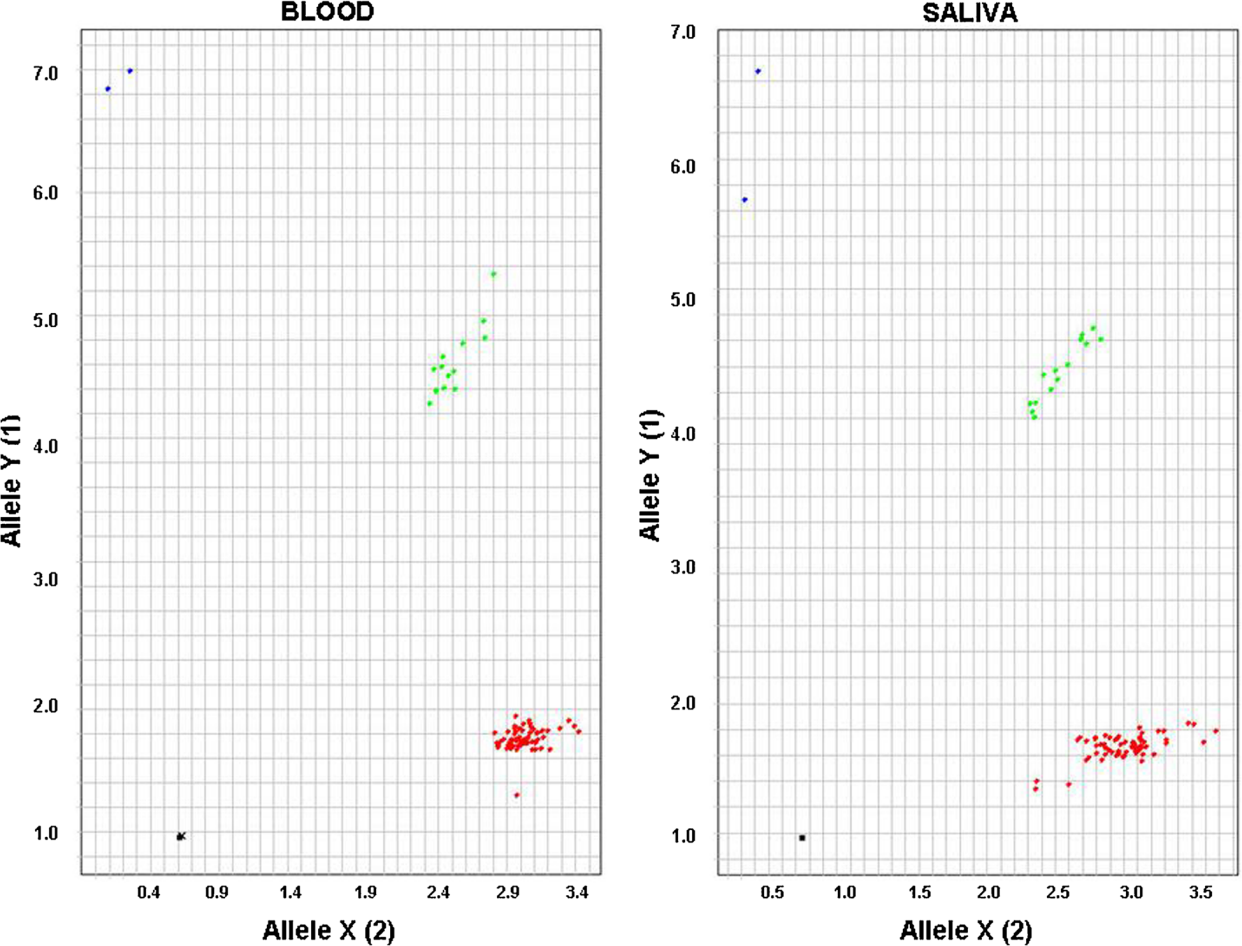
Example of Taqman Allelic Discrimination Plots for SNP rs3924194 on DNA from blood (right) and saliva (left).

Genotype Call Rates (GCR) for the Taqman assays were expressed as the proportions of DNA samples generating results in each SNP assay and for the Illumina Beadchip assays these were the number of SNPs called per DNA sample (Tables 2 and 3). Call rates for blood DNA were all >99%, using both measures, and call rates for saliva DNA were comparable (>97%). Figure 1 shows an example Taqman genotype plots for SNP rs3924194 and illustrates that signal strength and cluster definition are very similar for DNA from both sources.

**Table 2:** Taqman genotype call rates (GCR)

**SNP**	**Sample call rate blood DNA**	**Sample call rate saliva DNA**
rs12642938	157/158	152/158
rs10028494	157/158	151/158
rs7435335	157/158	158/158
rs3924194	156/158	157/158
**Overall**	**627/632 (99.2%)**	**618/632 (97.8%)**

The number of samples successfully genotyped/the total number of samples attempted, for each SNP tested.

**Table 3:** Illumina genotype call rates (GCR)

**Sample**	**Mean SNP calls blood ** **DNA**	**Mean SNP calls saliva DNA**
1	316,954	315,527
2	316,057	310,761
3	317,271	316,657
4	314,863	312,943
**Overall**	**316,286/318,075 (99.4%)**	**313,972/318,075 (98.7%)**

The number of SNPs successfully genotyped/the total number SNPs common to both Illumina HumanHap370 and HumanHap550 chips (318,075) for the 4 samples tested.

As a further test of the quality of the DNA, we also examined the genotype concordance between duplicate DNA samples in which both gave a result - a measure of the accuracy and hence reproducibility of the genotype calling. Concordance rates were high - for Taqman genotyping they were all >99% and for Illumina beadchip genotyping they were >99% for blood DNA and 100% for saliva DNA (Table 4 and 5).

**Table 4:** Taqman genotype concordance

**SNP**	**Matched blood DNA**	**Matched saliva DNA**	**Matched blood/saliva DNA**
**rs12642938**	78/78	75/75	73/74
**rs10028494**	78/78	75/75	74/74
**rs7435335**	78/78	79/79	77/78
**rs3924194**	77/77	78/78	76/76
**Overall**	**311/311 (100%)**	**307/307(100%)**	**300/302 (99.3%)**

The number of concordant results/the number of samples with 2 genotype calls for the 4 SNPs tested in 79 samples.

**Table 5:** Illumina genotype concordance

**Sample**	**Matched blood DNA**	**Matched saliva DNA**	**Matched blood/saliva DNA**
1	306,737/307,301 (99.8%)	313,721/313,811 (100%)	309,996/310,351 (99.9%)
2	314,603/314,646 (100%)	316,572/316,579 (100%)	314,421/314,479 (100%)
3	315,719/315,732 (100%)	315,688/315,736 (100%)	315,675/315,707 (100%)
4	303,915/304,196 (99.9%)	313,665/313,721 (100%)	309,613/309773 (99.9%)
**Overall**	**99.9%**	**100%**	**100%**

The number of concordant results/the total number of SNPs with 2 genotype calls for the 4 samples tested on Illumina chips.

During the course of this study, Illumina changed their chip format such that the saliva DNA samples were genotyped on HumanHap300 Duo BeadChips (total 318,511 SNPs) while the matched blood DNA samples were genotyped on HumanHap550 Duo BeadChips (total 555,352 SNPs). We thus restricted our analyses to the 318,075 SNP assays common to the two formats (Tables 3 and 5).

## Discussion

These results demonstrate that saliva DNA was of comparable quality to blood DNA. A valid concern, prior to this study, was that saliva may contain significant proportions of DNA from oral bacteria and/or food. If this had been the case, the true concentration of human DNA added to each assay would have been below the Picogreen calculated concentration and overall call rates would have been proportionately reduced. We observed negligible evidence of any such problem in our assays, although we did observe evidence of greater protein contamination and DNA fragmentation in the saliva DNA.

This study has some limitations. The change in Illumina chip format made our study design sub-optimal, however, the very high concordance rates we observe between saliva and blood DNA despite the change in format demonstrate that this issue, which was beyond our control, has not affected the conclusions of our study. The actual volume of saliva received was not recorded. This value would have provided an estimate of patient compliance with manufacturers’ instructions for using the saliva collection kit and producing the required amount of saliva in the sample. However, the aim of this article was to establish whether DNA of an acceptable quality and quantity could be obtained to allow high-throughput genotyping on different platforms, which we have successfully demonstrated.

The results of this feasibility study have already informed the collection of saliva samples to increase recruitment into Pharmacogenetics of Breast Cancer Chemotherapy (PGSNPS) study. A total of 444 saliva samples have been collected and 19% of all the DNA samples analysed from this study to date have been saliva derived.

Individual saliva sampling kits are more expensive than blood sampling kits however this cost has to be counter-balanced by the increased inconvenience to the patient and the cost of trained staff required to obtain blood samples. In addition commercial extraction of DNA from saliva is cheaper than from blood (Gen-Probe extraction costs for blood £8/sample and for saliva £5.10/sample).

## Conclusions

Although DNA yield is approximately 2-fold lower from saliva than from blood and other measures indicate slightly lower quality DNA, the key measure of genotyping quality is comparable on both Taqman and Illumina genome-wide beadchip arrays platforms. Saliva collection is less invasive than blood collection and participants who followed printed instructions, without supervision, provided useful quantities of DNA. The collection of saliva-derived DNA could substantially improve recruitment to translational studies in clinical trials, reduce costs and logistical problems associated blood collection within multicentre studies.

## Competing interests

The authors declare that they have no competing interests.

## Authors' contributions

JEA and SI dealt with ethics applications and recruitment to the study. JEA, MM, CC, PDPP and AD designed the study. MM and CL planned and performed the Taqman genotyping. IS and JEA performed chip genotyping. RR, MM and JEA completed the data analysis. AD, HME, CC, MM and JEA drafted the manuscript. All authors read and approved the final manuscript.

## Pre-publication history

The pre-publication history for this paper can be accessed here:

http://www.biomedcentral.com/1755-8794/5/19/prepub

## Supplementary Material

Additional file 1 **Figure 1.** Primers and probes for Taqman assays. Figure 2 Assessment of fragmentation of 3 matched blood and saliva derived DNA samples. Table 1 Ratio of absorbance at 260/280nm for 10 random, matched samples. Click here for file
