# Supplementary material for: Saliva samples are a viable alternative to blood samples as a source of DNA for high throughput genotyping
Source: BMC Med Genomics. 2012 May 30;5:19. doi: 10.1186/1755-8794-5-19 (PMC3497576; doi:10.1186/1755-8794-5-19)

**Additional files**

**Supplementary Figure 1**

**Primers and probes for Taqman assays**

**rs12642938**

Forward primer: ACTCCCAGTTATATACCCAACAGATATGTATAAAT

Reverse primer: TGTCTTCATTTTATGATTAATAGATATTTGGGTTGCT

VIC probe: CAATTTTTGGACAATATTGAT

FAM probe: ATTTTTGGACAGTATTGAT

**rs10028494**

Forward primer: CCAATGGAGGAAGCAGAAGGAAATA

Reverse primer: ACAAATACACAAAGGTCACTGGCTTA

VIC probe: CAAACAGAAGGAGGCCAG

FAM probe: AACAGAAGGCGGCCAG

**rs7435335**

Forward primer: CCAAATTCAGCAAGATACAATTTTGAGTTTTT

Reverse primer: TCAGCATAGAAATGAAGAATCTGTTGGT

VIC probe: AACAGCTGTGTTTTT

FAM probe: TCAACAGCTATGTTTTT

**rs3924194**

Forward primer: TTCCTGCCTGATAAAGCTTTCTTGT

Reverse primer: CAGTGTTTAACTTTTATTTGCTACATCAGTGT

VIC probe: CTCTTCCAACTTTTGC

FAM probe: TCTTCCAAGTTTTGC

**Supplementary Table 1**

**Ratio of absorbance at 260/280nm for 10 random, matched samples**

| **Sample** | **A260/280 Ratio** | |
| --- | --- | --- |
|  | **Saliva** | **Blood** |
| **1** | 1.71 | 1.57 |
| **2** | 1.55 | 1.61 |
| **3** | 1.66 | 1.78 |
| **4** | 1.63 | 1.75 |
| **5** | 1.34 | 1.64 |
| **6** | 1.55 | 1.78 |
| **7** | 1.68 | 1.81 |
| **8** | 1.43 | 1.77 |
| **9** | 1.38 | 1.63 |
| **10** | 1.66 | 1.73 |
| **Mean** | **1.56** | **1.71** |

**Supplementary Figure 2**

**Assessment of fragmentation of 3 matched blood and saliva derived DNA samples**


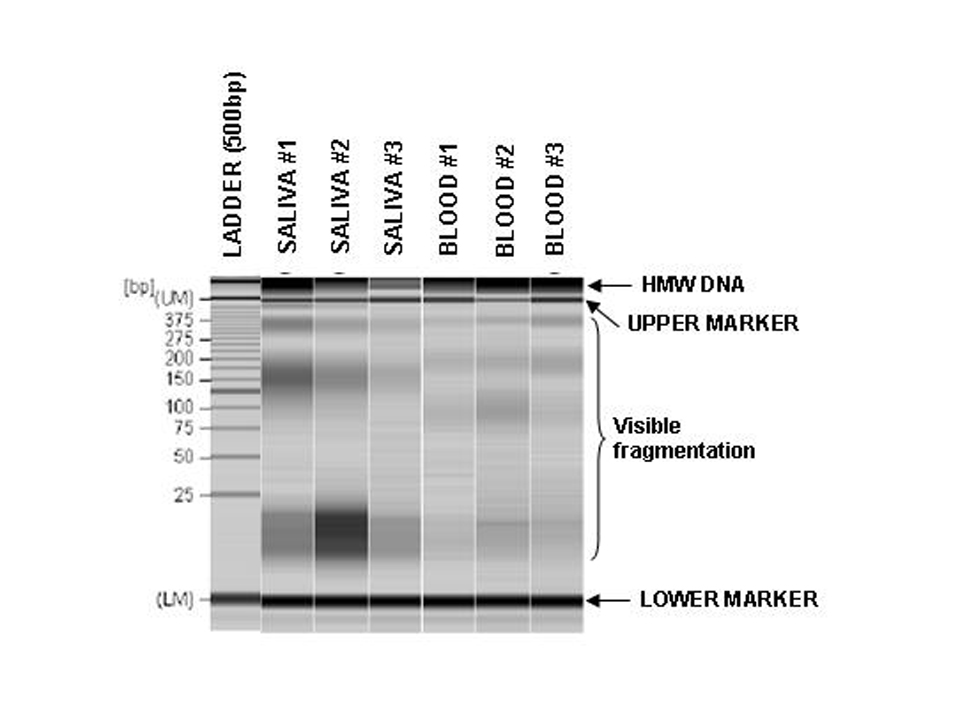

Supplement: Additional file 1 — Figure 1. Primers and probes for Taqman assays. Figure 2 Assessment of fragmentation of 3 matched blood and saliva derived DNA samples. Table 1 Ratio of absorbance at 260/280nm for 10 random, matched samples. [file 1755-8794-5-19-S1.doc]
